# Predicting Adult Hospital Admission from Emergency Department Using Machine Learning: An Inclusive Gradient Boosting Model

**DOI:** 10.3390/jcm11236888

**Published:** 2022-11-22

**Authors:** Dhavalkumar Patel, Satya Narayan Cheetirala, Ganesh Raut, Jules Tamegue, Arash Kia, Benjamin Glicksberg, Robert Freeman, Matthew A. Levin, Prem Timsina, Eyal Klang

**Affiliations:** 1 Mount Sinai Health System, New York, NY 10017, USA; 2 Department of Anesthesiology, Perioperative and Pain Management, Mount Sinai Hospital, New York, NY 10017, USA

**Keywords:** machine learning, healthcare, clinical decision support

## Abstract

Background and aim: We analyzed an inclusive gradient boosting model to predict hospital admission from the emergency department (ED) at different time points. We compared its results to multiple models built exclusively at each time point. Methods: This retrospective multisite study utilized ED data from the Mount Sinai Health System, NY, during 2015–2019. Data included tabular clinical features and free-text triage notes represented using bag-of-words. A full gradient boosting model, trained on data available at different time points (30, 60, 90, 120, and 150 min), was compared to single models trained exclusively at data available at each time point. This was conducted by concatenating the rows of data available at each time point to one data matrix for the full model, where each row is considered a separate case. Results: The cohort included 1,043,345 ED visits. The full model showed comparable results to the single models at all time points (AUCs 0.84–0.88 for different time points for both the full and single models). Conclusion: A full model trained on data concatenated from different time points showed similar results to single models trained at each time point. An ML-based prediction model can use used for identifying hospital admission.

## 1. Introduction

Emergency departments (ED) are overcrowded in the United States and internationally, hindering patient care and system efficiency. ED overcrowding has been associated with increased mortality and morbidity, longer wait times, and length of stay. Overcrowding has also increased hospital expenses and generated poorer patient perceptions of care [1,2,3,4].

The ED admission process is aimed to expedite patient disposition, as EDs are becoming overcrowded. Typically, ED admission is started by a triage nurse that performs the triage process. The nurse records the patient’s demographic data and measures vital signs. The nurse also records the patient’s visit reason in a free-form text note [4]. Other clinical data accumulate as the patient is in the ED. This mainly includes laboratory results that can infer essential clues to the patient’s clinical condition. For example, leukocytosis (WBC) may signal an infection, and increased troponin may signal myocardial infarction.

In hospitals with EHRs, where patient data are recorded at the point of care, EHR data can be utilized to generate short-term predictions of hospital admissions and thus bed demand. These would help control teams, responsible for allocating beds, to make best use of available capacity and reduce cancellations of elective admissions [5]. Secondly, patients can anticipate hospitalization, which could increase patient satisfaction. Thirdly, it may have prognostic value as patients who need hospitalization are often the sickest and will benefit most from time-sensitive ED treatment [6].

Increases in digital electronic health records (EHR) data volume [7] are driving machine learning use in healthcare processes [8,9]. Previous works utilized ED data [10,11] for building models for predicting hospital admission.

It is important to note that triage is not the same as the hospital admission prediction (also known as patient disposition). ED data include multiple features that stream at different progressive time points. Thus, different predictions can be made depending on the time from the patient’s arrival at the ED. Yet, previous works presenting decision support tools for predicting disposition from the ED did not describe a full gradient boosting model that handles multiple time points. Such a model may be easier to train and implement than multiple models trained exclusively at each time point. However, it should be tested whether such a model remains stable, as it aggregates multiple time points.

This study aimed to develop an inclusive tabular–free–text gradient boosting model for predicting hospital admission and compare its results to multiple time points models.

## 2. Methods

### 2.1. Data Sources

The model is built using patient data from Mount Sinai Health System (MSHS), an urban health system in New York City. Emergency Department data were obtained from five hospitals within the MSHS in New York City: The Mount Sinai Hospital (MSH), located in East Harlem, Manhattan; Mount Sinai Morningside (MSM), located in Morningside Heights, Manhattan; Mount Sinai West (MSW) located in Midtown West, Manhattan; Mount Sinai Brooklyn (MSB) located in Midwood, Brooklyn; and Mount Sinai Queens (MSQ) located in Astoria, Queens. The data set was obtained from different sources using the Epic EHR software (Epic Systems, Verona, WI, USA) and aggregated by the Clinical Data Science team.

An institutional MSHS ethical board committee approval was granted for this retrospective study. The committee waived informed consent.

### 2.2. Study Design

**Figure 1:**
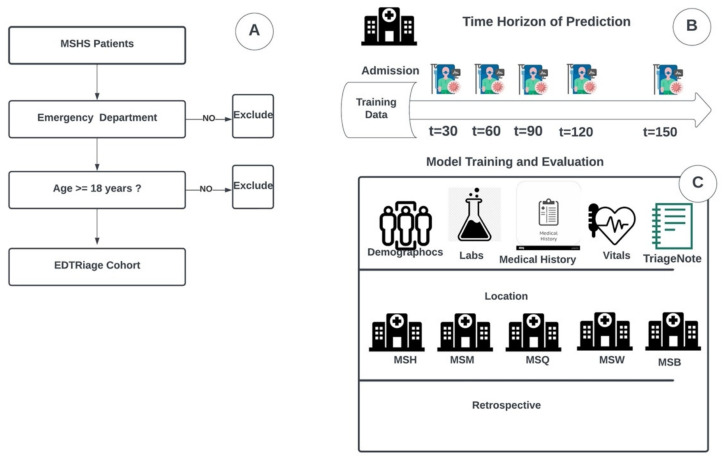
Study design and workflow. (**A**) Procedure for patient inclusion in our study. (**B**) Time horizon of prediction on multiple time frames after patient’s admission (**C**) Strategy and design of the experiments. Emergency Department patient data from the Mount Sinai Health System data warehouse were used to train and validate the XGBoost model. Data include demographic, lab, and vital signs. They also include triage notes from the emergency department written by nurses from all five hospitals: The Mount Sinai Hospital (MSH) located in East Harlem, Manhattan; Mount Sinai Morningside (MSM) located in Morningside Heights, Manhattan; Mount Sinai West (MSW) located in Midtown West, Manhattan; Mount Sinai Brooklyn (MSB) located in Midwood, Brooklyn; and Mount Sinai Queens (MSQ) located in Astoria, Queens.

Using the MSHS data warehouse, we identified patients who presented to our ED between January 2015 to December 2019. We extracted tabular clinical and demographic data from this cohort, including all free-text triage notes. Figure 1A presents the data collection method.

For all ED patients within the extracted cohort, the models were designed considering tabular EHR data and triage notes. The single models were developed on five different time points data (30, 60, 90, 120, and 150 min), and the full model was developed using all time points data concatenated. All models’ testing sets results were compared using confusion matrix metrics and the area under the receiver operating characteristic (ROC) curves (AUC).

### 2.3. Study Population

We retrospectively included patients who presented to the ED between 2015 and 2019. All patients over 18 years of age and admitted to the EDs of five MSHS hospitals. We excluded all patients younger than 18. We also excluded erroneously created or duplicated patient records.

### 2.4. Study Data

We extracted clinical and demographic data, including sex and age, hospital facility, admission time, vital signs, and laboratory results, as described in Figure 1C. Within the same data source, we also extracted all triage notes. Upon a patient’s arrival in the Mount Sinai ED, the first clinical documentation recorded in a patient’s chart is a triage note consisting of an abbreviated patient history written by a triage nurse. All included recorded data was time stamped.

### 2.5. Outcome Definition

The primary outcome was a prediction of hospitalization. The average disposition time from ED presentation is usually ≥3 h. This model can reduce hospitalization time and predict hospitalization starting from 30 min of patients’ admission to the ED.

### 2.6. Model Development

**Figure 2:**
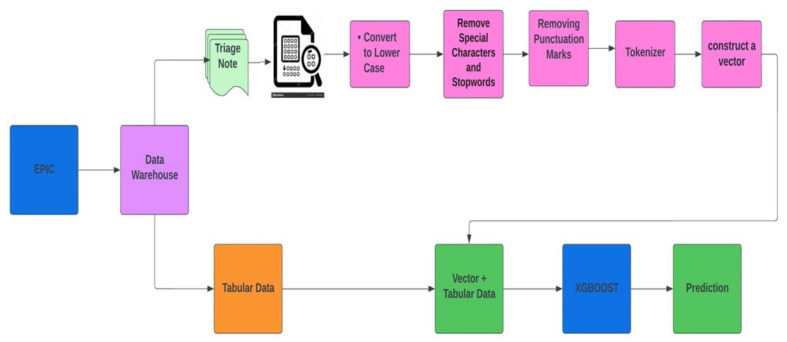
Diagram of how the XGBoost model was developed with a concatenation of the BOW model representing free-text triage notes and tabular structured EHR data.

For this study, we selected Extreme Gradient Boosting (XGBoost) implementation of gradient boosting decision trees [12]. The XGBoost algorithm provided robust prediction results through an iterative process of prediction summation in decision trees that fit the residual error of the prior ensemble. Both tabular and free-text data were used to build the model to predict patients’ admission from the ED. Table 1 presents the list of tabular features used in the models. The prediction is updated every 30 min from the patient’s ED arrival time. Model development and how the dataset moves from epic to XGBoost model for prediction are described with an architecture diagram in Figure 2.

The bag-of-words (BOW) model was implemented on triage free-text notes. BOW is a representation that turns arbitrary text into fixed-length vectors by counting word frequencies, and this process is often referred to as vectorization. A statistical classifier is then trained to classify each paragraph based on word frequency and number. To implement the BOW model, we preprocess the data by converting text to lower case and removing all non-word characters and punctuations. The BOW Vector and tabular EHR data were combined using sparse vector representation.

Multiple models were developed to ensure its performance in the form of Experiments. In Experiment 1, multiple single models were developed and trained on multiple time points data with 30, 60, 90, 120, and 150 min timeframes. In Experiment 2, we developed a full model trained on the concatenated entire time points data and tested on the multiple time points (30, 60, 90, 120, and 150 min). This was done by concatenating the rows of data available at each time point to one data matrix for the full model, where each row is considered a separate case. Thus, at each time point, the full model gives predictions based on the data available at that time.

### 2.7. Models Training and Testing

Data were split chronologically. Data from the years 2015–2018 were used for training and validation, and the 2019 data were used for testing. The default XGBoost hyper-parameters were used for all the models: eta = 0.3, max depth = 3. We set n_estimators = 200. The XGBoost model handled imputations of missing values. Scale balancing of the XGBoost was set to the default scale pos weight = 1.

### 2.8. Models Interpretation

We evaluated the performance of the full vs. single models for each time point (30, 60, 90, 120, and 150 min). Single feature analysis was also performed for the structured variables. SHapley Additive exPlanations (SHAP) summary plots were constructed to assess the full XGBoost model feature importance. Finally, using the full dataset, we compared a structured data only model to a free-text-only model.

### 2.9. Statistical Analysis

All development and statistical analysis were done using Python (Version 3.6.5). Continuous variables are reported as the median, with the spread reported as the Interquartile range (IQR). Categorical variables are reported as percentages. Categorical variables were compared using the χ2 test, and continuous variables were compared using Student’s t-test. Statistical significance was established at a two-sided *p*-value of *p* < 0.05.

We constructed receiver operating characteristic (ROC) curves for all models and evaluated the AUC. We determined sensitivity (recall), specificity, and precision (positive predictive value, PPV) for a default cut-off probability of 0.5. Confusion matrix values (true positive, false positive, true negative, false negative) are also reported for this cut-off probability.

## 3. Results

### 3.1. Cohort Characteristics

1,043,345 ED visits in five years (2015–2019) were included. Of those, 19.3% were hospitalized (*n* = 201,520). The median time from ED entrance to ED disposition time was 194 min (IQR 113–314 min). Table 2 represents the cohort’s characteristics. Patients admitted to the hospital had an average age of 64. They were more likely to have higher systolic blood pressure, higher respiratory rate, and higher heart rate (Table 1).

### 3.2. Experimental Results

To evaluate an inclusive tabular-free-text gradient boosting model which predicts hospitalization, we have tested two experiments on multiple progressive time points.

#### 3.2.1. Experiment 1

**Figure 3:**
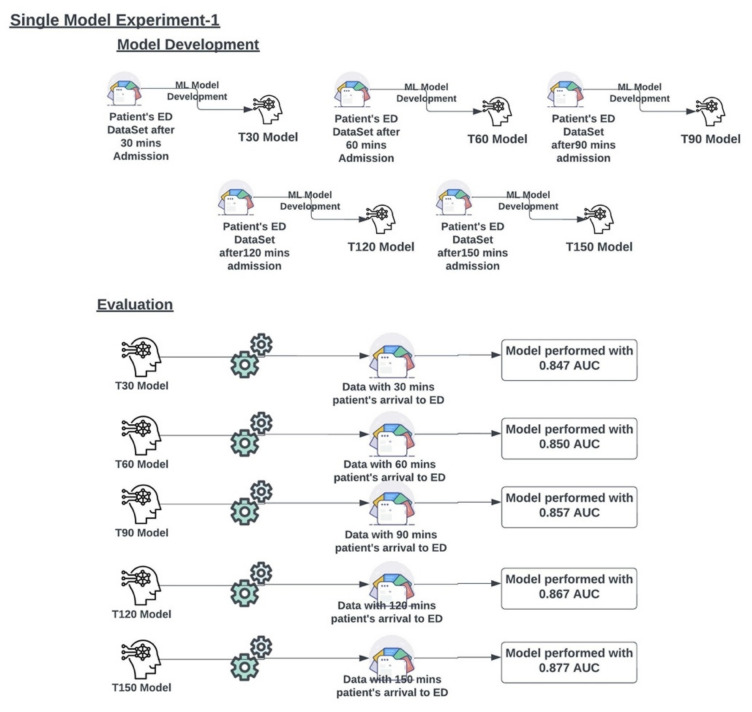
Multiple single models were developed on multiple time point data sets and evaluated on multiple time point data to check their performance.

**Figure 4:**
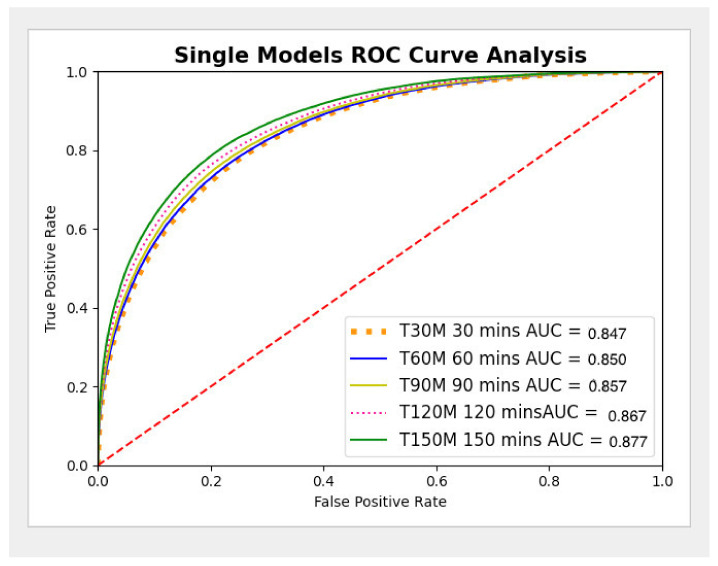
Single Model ROC Curve analysis.

Five single models (T30, T60, T90, T120, and T150) were developed on the ED dataset at multiple time points (30, 60, 90, 120, and 150 min from presentation to the ED), as described in Figure 3. The AUC metrics for experiment 1 were generated for each single model (T30, T60, T90, T120, and T150) and are presented in Figure 3 and the ROC curves are presented in Figure 4. The metrics of the single models are presented in Table 3.

#### 3.2.2. Experiment 2

**Figure 5:**
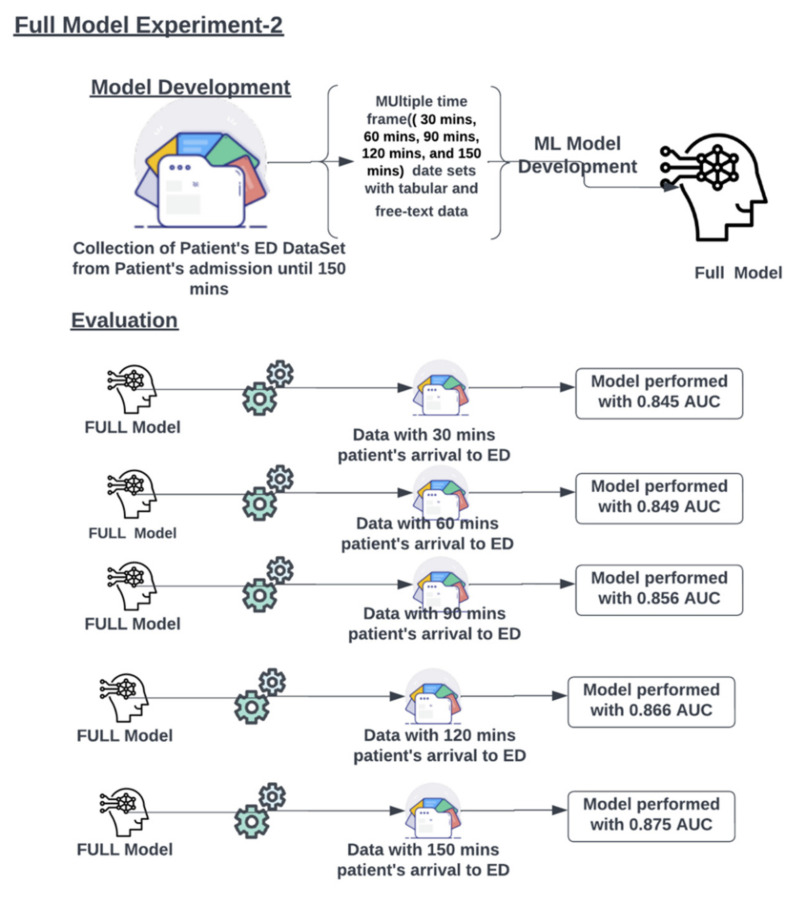
The full model was developed on single large data sets and evaluated on multiple time point data to check its performance. The figure presents the AUCs of the model at the different time points.

**Figure 6:**
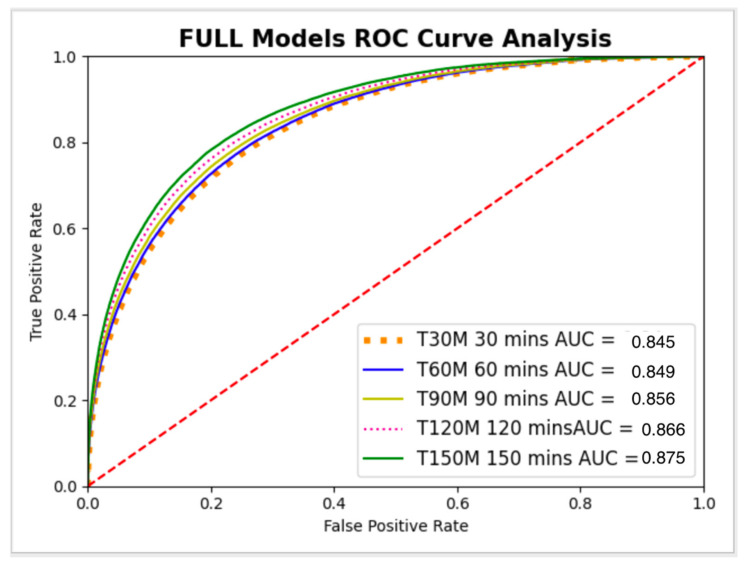
Full Model ROC Curve analysis.

The full model is developed using the ED patients dataset with all-time points data concatenated as one dataset, as presented in Figure 5. The full model was tested individually on multiple progressive time point datasets (30, 60, 90, 120, and 150 min from ED presentation), as described in Figure 6. The metrics for the full model on different time points are presented in Table 4.

For all time points, AUC confidence intervals (CI) overlapped between the single models and the full model. Thus, no significant statistical difference was shown (Table 5).

For the full model, for all time points, we’ve also compared structured data only model vs. free-text data only model. For structured data only, the AUC was 0.84; for free-text data only, the AUC was 0.79.

We also trained the full model (tabular + text) without the ESI feature. For this model, the AUC was 0.86. This is comparable to the full model (tabular + text) with the ESI feature, which showed a similar AUC of 0.87.

### 3.3. Single Feature Analysis

In the single feature matrix analysis, the tabular variables with the highest AUC were Age (AUC 0.726), followed by ESI (AUC 0.722), sodium (AUC 0.621), and calcium (AUC 0.618). The single feature analysis is presented in Table 6.

### 3.4. SHAP Plot Analysis:

**Figure 7:**
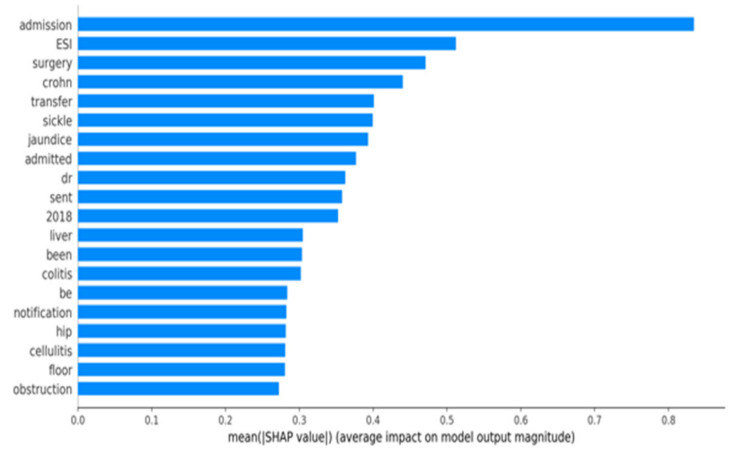
SHAP Plot of the full model at all time points.

Figure 7 shows the SHAP plot of the full model analyzed at all time points. Our analysis of ED notes suggests that certain features are highly associated with hospitalization. Based on the SHAP graph, the top three terms were “admission”, “surgery”, and “crohn”. Similarly, the structured feature “ESI”, which is nurse assessment of severity (scale 1–5), was also a high predictor of hospital admission.

**Figure 8:**
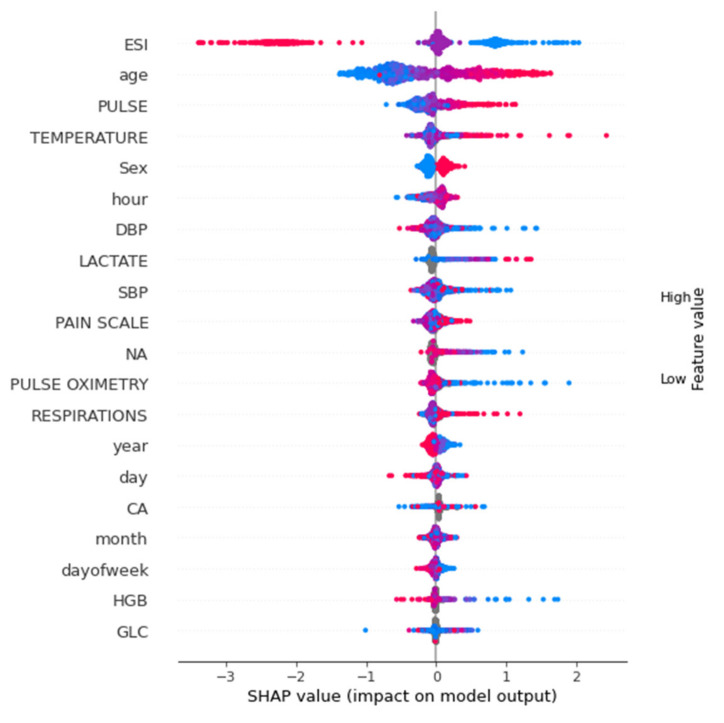
SHAP summary plot of tabular features.

Figure 8 shows the SHAP summary plot of the tabular data full model analyzed at all time points. For tabular data-only analysis, ESI, age, pulse, temperature and sex were the features with the highest importance.

## 4. Discussion

In this study, we developed a machine learning model based on free-text triage notes and EHR tabular data to predict hospital admission from the ED. For this task, a full gradient boosting model, trained on the entire structured and free-text data at different time points, showed stability compared to multiple single models trained on various time-frame points. Using a full model may be easier to train and implement.

Several previous works presented models for the prediction of hospital admission. For example, Hong et al. compared gradient boosting (XGBoost) to deep neural networks for predicting hospital admission with demographics and triage features. Both models showed similar results [10]. Their models showed AUCs of 0.87, which is comparable to the current AUC of 0.85–0.88. However, Hong et al. used previous patient data (medication, past medical history), while we used more current HER data (labs). Their approach has benefits, although it requires the patient to be seen in the same health system before to have these data recorded and also to maintain an updated hashed data lake for instant access for each patient.

Raita et al. predicted both hospitalization and critical care outcomes, and their features included demographics, vital signs, chief complaints, and comorbidities. They’ve shown that machine learning models (lasso logistic regression, random forest, gradient boosting, neural networks) outperformed logistic regression [13]. Their neural network model showed similar results to our model (AUC 0.86) while again using previous medical history data (comorbidities). Unlike the previous examples that evaluated a single model at triage time, Lee et al. used logistic regression to predict admission at three different positions. They built single models using three data sets (demographics, triage vitals, and laboratory results) [14]. Lee’s model predicted different disposition endpoints, including ICU, but general practice and observation units showed similar AUCs to our model (0.89, 0.86). Barak et al. also evaluated single logistic regression models at three progressive time stops, using demographics, triage, and laboratory data [15]. Barak’s et al. model showed an AUC of 0.97, which is higher than all other cited models and our model. We cannot explain this difference based on available data.

While the previous studies evaluated only structured data, several studies used free-text. For example, Lucini et al. utilized provider notes available several hours after triage to train several machine learning models (for example, random forest and logistic regression) [16]. Sterling et al. used the single data source of triage notes using neural network regression models with bag-of-words (BOW) [4]. Sterling’s model, which utilized triage notes as a single feature, showed an AUC of 0.73.

Our previous study trained a single triage data gradient boosting model (demographics, vital signs, and triage notes) to predict admission to the neurosurgical intensive care unit [17].

In the current study, we evaluated the use of one full gradient boosting (XGBoost + BOW) model at multiple progressive time points (every 30 min after the patient presented to the ED) using both structured and free-text data. We compared the full model to single models trained at each time position.

While the full model demonstrated satisfactory results and performed well within 30 min of the patient’s admission to ED, by using continually aggregated data from the ED, the models showed increased sensitivity of 15% from 30 min to 150 min while maintaining the same PPV. Such a solution is simple to train and easy to deploy.

A machine learning model like the one presented could be used by hospital care experts and clinical stakeholders, such as ED clinicians and nursing managers, to identify patients that might need hospitalization early in the ED triage process. This could also allow providers to administer timely care specialists, accelerate patient movement into the hospitals, and potentially reduce ED boarding time.

Implementing the model presented here could be a clinical decision support tool that identifies patients requiring hospital admission and delivers a notification to the specialized care team. In such implementation, selecting an optimal alert threshold necessitates a careful evaluation of model performance and likely depends on multiple factors, including healthcare institution needs, clinical stakeholder preferences, and hospital resource availability.

It should be noted that ESI showed a high AUC in a single feature matrix. To compare the functional value of our model to triage by humans (ESI), we evaluated the AUC of the full model without the ESI feature. This showed an AUC of 0.86. Thus, although ESI by itself has high prediction capability, by utilizing multiple features, the model can “overcome” the loss of ESI as a feature.

To summarize, in this study, we used ED features (demographics, vital signs, ESI, triage note, and laboratory data) to develop a model for predicting hospital admission at different time points from ED presentation. Our model is based on the gradient boosting algorithm, with a BOV approach for free-text notes. We compared a single full model, trained at all time points, to multiple single models, trained at each time point. We have shown that for each time point the full model achieves similar results to the single models. Thus, potentially making it easier to be trained and implemented.

Our study has several limitations. First, this is a retrospective study. Second, while we experimented with the widely used gradient boosting algorithm, other approaches, such as deep learning transformer models, may show better results. Third, it is important to note that each hospital has its own admission policy. Thus, our model serves as a proof of concept, and cannot be implemented as is in a new site, but needs to be adjusted according to the site’s setting and policy. Fourth, our model is not intended for the triage setting, as it uses out-of-triage features, mainly laboratory data.

In conclusion, using tabular and free-text data, a model trained at different time points can predict hospital admission from the ED. A full model trained on data from all time points showed similar results to single models trained exclusively at different time points. This may simplify the training and implementation of such a model.

## Figures and Tables

**Table 1:** List of tabular features used in the models.

Demographics and Presentation	Vital Signs	Laboratory
Sex	Temperature	WBC
Age	Respirations	HGB
ESI	PainScale	PLT
Month	PulseOximetry	NEUT
Year	Pulse	LYMPH
Day	SystolicBP	CR
Hour	DiastolicBP	BUN
DayOfWeek		GLC
		NA
		K
		CHLORIDE
		CALCIUM
		LACTATE
		ALT
		AST
		BILIRUBIN
		ALK PHOS
		ALBUMIN
		CRP
		D-DIMER
		BNP
		CPK
		TROPONIN

**Table 2:** Presents clinical and demographic characteristics of the patients’ cohort, stratified by admission to the emergency department.

	Discharged (*n* = 841,825, 80.7%)	Hospitalized (*n* = 201,520, 19.3%)	*p* Value
Age, median (IQR), y	43.0 (29.0–59.0)	64.0 (49.0–77.0)	<0.001
Female, N. (%)	465,178 (55.3)	103,030 (51.1)	<0.001
SBP, median (IQR), mmHg	130.0 (118.0–145.0)	134.0 (117.0–153.0)	<0.001
DBP, median (IQR), mmHg	76.0 (68.0–85.0)	74.0 (65.0–85.0)	<0.001
Heart rate, median (IQR), beats/min	82.0 (73.0–93.0)	87.0 (75.0–101.0)	<0.001
Temperature, median (IQR), Celsius	36.7 (36.4–36.9)	36.7 (36.4–37.0)	<0.001
Respirations, median (IQR), breaths/min	18.0 (17.0–19.0)	18.0 (18.0–20.0)	<0.001
O^2^ saturation, median (IQR)%	98.0 (97.0–99.0)	98.0 (96.0–99.0)	<0.001
WBC, median (IQR), ×10^3^/uL	7.8 (6.1–9.9)	8.8 (6.6–11.9)	<0.001
NEUT, median (IQR), ×10^3^/uL	5.0 (3.6–7.0)	6.2 (4.2–9.3)	<0.001
HGB, median (IQR), g/dL	13.1 (11.9–14.3)	12.3 (10.5–13.8)	<0.001
BUN, median (IQR), mg/dL	13.0 (10.0–17.0)	17.0 (12.0–27.0)	<0.001
Cr, median (IQR), mg/dL	0.8 (0.7–1.0)	0.9 (0.7–1.4)	<0.001

**Table 3:** Derived metrics of the single models.

Minutes	True Positive	False Positive	True Negative	False Negative	Sensitivity	Specificity	Precision (PPV)
30	21,221	9224	255,897	39,829	0.348	0.965	0.697
60	21,887	9252	255,869	39,163	0.359	0.965	0.703
90	23,247	9575	255,546	37,803	0.381	0.964	0.708
120	25,172	9731	255,390	35,878	0.412	0.963	0.721
150	27,473	10,367	254,754	33,577	0.450	0.961	0.726

**Table 4:** Derived metrics of the full model at multiple time points.

Minutes	True Positive	False Positive	True Negative	False Negative	Sensitivity	Specificity	Precision (PPV)
30	19,352	7810	257,311	41,698	0.317	0.971	0.712
60	21,342	8850	256,271	39,708	0.350	0.967	0.707
90	23,000	9345	255,776	38,050	0.377	0.965	0.711
120	25,147	9991	255,130	35,903	0.412	0.962	0.716
150	27,708	11,197	253,924	33,342	0.454	0.958	0.712

**Table 5:** Comparison of full model and single models AUC confidence intervals (CI) at different time points.

	Full Model AUC	Single Models AUC
30 min	0.845 (95% CI: 0.843–0.847)	0.847 (95% CI:0.845–0.849)
60 min	0.849 (95% CI: 0.848–0.851)	0.850 (95% CI:0.849–0.852)
90 min	0.856 (95% CI: 0.855–0.858)	0.857 (95% CI:0.855–0.858)
120 min	0.866 (95% CI: 0.864–0.867)	0.867 (95% CI:0.865–0.868)
150 min	0.875 (95% CI: 0.874–0.877)	0.877 (95% CI:0.876–0.879)

**Table 6:** Tabular variables with the highest AUC contributions for admission to the hospital from ED.

Feature	AUC
Age	0.726
ESI	0.722
Na^+^	0.621
Ca^++^	0.619
K^+^	0.618
Hgb	0.611
Glucose	0.603
Lactate	0.603
Cl^-^	0.602
White Blood Cell Count	0.595
Neutrophil Count	0.595
Platelet Count	0.594
Lymphocyte Count	0.591
Pulse Rate	0.591
Sytolic BP	0.581
